# Experimental Examination of Intraspecific Density-Dependent Competition during the Breeding Period in Monarch Butterflies (*Danaus plexippus*)

**DOI:** 10.1371/journal.pone.0045080

**Published:** 2012-09-12

**Authors:** D. T. Tyler Flockhart, Tara G. Martin, D. Ryan Norris

**Affiliations:** 1 Department of Integrative Biology, University of Guelph, Guelph, Ontario, Canada; 2 CSIRO Ecosystem Sciences, Dutton Park, Queensland, Australia; California State University Fullerton, United States of America

## Abstract

A central goal of population ecology is to identify the factors that regulate population growth. Monarch butterflies (*Danaus plexippus*) in eastern North America re-colonize the breeding range over several generations that result in population densities that vary across space and time during the breeding season. We used laboratory experiments to measure the strength of density-dependent intraspecific competition on egg laying rate and larval survival and then applied our results to density estimates of wild monarch populations to model the strength of density dependence during the breeding season. Egg laying rates did not change with density but larvae at high densities were smaller, had lower survival, and weighed less as adults compared to lower densities. Using mean larval densities from field surveys resulted in conservative estimates of density-dependent population reduction that varied between breeding regions and different phases of the breeding season. Our results suggest the highest levels of population reduction due to density-dependent intraspecific competition occur early in the breeding season in the southern portion of the breeding range. However, we also found that the strength of density dependence could be almost five times higher depending on how many life-stages were used as part of field estimates. Our study is the first to link experimental results of a density-dependent reduction in vital rates to observed monarch densities in the wild and show that the effects of density dependent competition in monarchs varies across space and time, providing valuable information for developing robust, year-round population models in this migratory organism.

## Introduction

A central tenet of population ecology is to identify the factors that regulate population growth [1]–[3]. However, we know surprisingly little about how wild populations are regulated by density dependent processes despite the importance of estimating density dependence for developing predictive models. The fact that density dependence can operate at different stages of the life cycle [4]–[6] suggests that assessing density dependence is particularly difficult for migratory species because populations may be regulated at multiple stages of the life cycle that occur in geographically separated regions [7]. In North America, monarch butterflies (*Danaus plexippus*) show a variety of life history strategies [8] that are expected to influence population dynamics (e.g. [9], [10]). Eastern populations undertake a long-distance migration and re-colonize the breeding distribution over successive generations [11]–[13] and population density varies within seasons [10], [14], [15], among years [10], [16], [17], and between different regions on the breeding grounds [10], [15], [16]. As a result, if density-dependent effects operate on the breeding grounds then quantifying how variation in density influences growth, survival or reproductive rates could be used to predict changes in population growth rates that are spatially and temporally dependent.

Reproductive female monarch butterflies typically lay one egg per plant [18], [19] but several females visiting the same plant could result in numerous eggs, suggesting that competition for larval host plants among adult females could reduce per capita reproductive output whereas direct competition among larvae could reduce individual survival. Larval competition could also operate indirectly through reduced growth and body condition that influence lifespan and ultimately future lifetime fecundity during adulthood [4], [20]. In this study, we experimentally manipulated adult female density and egg density to examine the potential effects of intraspecific competition on (1) adult female egg laying rate and (2) larval growth and survival rate. We then applied our experimental results to previously reported monarch larval densities in North America to estimate the effect of density-dependent competition in different breeding regions and during different periods of the breeding season.

## Methods

### Plants

We grew tropical milkweed (*Asclepias curassavica*) in commercial medium for both feeding and experimental purposes. Milkweed seeds were sprouted in growth chambers (500 mol light, 28°C, 80% RH, 18L:6D) until bearing 2–4 leaves and then transferred to larger trays until they were approximately 15 cm tall, at which time they were moved to a single glasshouse. Single plants were transferred to 10 cm-wide pots (feeding stock) while groups of 10 plants were transferred to 35 cm-wide pots to be used in experiments (see below). Milkweed was watered daily with distilled water and fertilized approximately weekly. To reduce the impact of Thysanoptera, we sprayed pressurized water against leaves and applied the predatory mite biocontrols *Amblyseius swirskii*, *A. cucumeris*, and *Hypoaspis aculeifer*. The glasshouse with the milkweed was maintained under ambient light conditions at 29°C during the day and 23°C at night.

### Breeding Stock

In a separate glasshouse, we maintained our monarch butterfly breeding stock and conducted all experiments. Throughout the experiment, temperature was maintained at 22°C during darkness and 28°C during daylight hours. The photoperiod cycle followed ambient light conditions until August 10, 2010 when it was switched to 16L:8D to reduce the chance of monarchs entering reproductive diapause [21]. We kept humidity between 75% and 100% in the monarch glasshouse to reduce desiccation and infertility of monarch eggs [22].

We provided tropical milkweed to 12 female and 12 male wild-caught monarchs from Guelph, Ontario (43.5°N, 80.2°W), that constituted our initial breeding stock and produced the adult females that were used for the density-dependent experiments. Larvae were raised on potted milkweed plants until approximately 3^rd^ instar when they were moved to individual plastic containers where they were fed ad libitum with glasshouse-raised tropical milkweed and local, wild-grown common milkweed (*A. syriaca*). Adult butterflies were provided daily with 10% sugar-water solution in small platform feeders. Feeders were washed and sanitized with bleach solution approximately every 3 days. Males and females were housed together and provided with milkweed plants in the afternoon to induce mating.

### Egg Laying Density Experiment

On the day of the experiment, individual females were randomly selected and provided with a milkweed plant. Any of the females that laid an egg immediately were selected for the trials and each female was only used once during the study. We continued until we had the number of females needed for the replicate on that day. We provided 5 pots (10 plants per pot, 50 total plants; mean plant height of replicates = 45.8 cm, SD = 7.7 cm) to 1 (*n* = 7), 4 (*n* = 5), 8 (*n* = 4) or 16 (*n* = 4) adult females in a 4 m^3^ enclosure for 4 hours. We chose the lowest adult density to imitate a situation with little perceived competition that may influence laying behaviour [23] because monarch butterflies lay approximately 50 eggs per day in captivity [20], [24] and normally lay one egg per plant [18]. At the end of the experiment we counted the number of eggs and divided by the number of females to calculate the mean per capita egg laying rate. We used the mean percent cloud cover recorded at the start and end of the experiment as well as the mean age since eclosion of all adults used in the replicate to account for how these factors influence laying rate [20], [24].

### Larval Density Experiment

We arranged densities of 1, 5, 10, 20, 35, and 50 eggs on 10 milkweed plants (replicate mean height = 46.9 cm, SD = 11.0 cm) by removing excess eggs after the egg laying experiment was complete until the number of eggs matched the required experimental larval density. The order of larval density treatments was not randomly assigned but there was no correlation between initiation date and replicate density (r = −0.09, P = 0.58). This range of densities (0.1 to 5 eggs per milkweed plant) was chosen because it encompassed a range of egg densities observed in the wild (range = 0−2.8 eggs/plant: [11], [25], [26]). Each trial was set in a netted enclosure (0.5 m×0.5 m×1.3 m). We measured (nearest 0.01mm; starting day of hatch), weighed (nearest 0.001g; starting two days after hatch), and recorded the larval instar of up to 10 individuals in each enclosure every 1–3 days. Because we only measured up to 10 individuals in the higher density treatments we did not make behavioral observations or record the stage of mortality. We weighed and measured each pupa 48 hours after formation, recorded the day that butterflies eclosed and, 24 hours after eclosion, weighed each butterfly and measured the forewing length for individuals that were not deformed. At the end of the experiment, we visually estimated the proportion of food resources remaining for each replicate.

### Applying Experimental Results to Natural Densities

We applied the proportional reduction in survival caused by density dependence found in our experiment to natural larval densities of monarchs recorded between 1997 and 2006 throughout the monarch range in eastern North America [27]. We used information from Lindsey et al. [15] who calculated larval density as the sum of 3^rd^, 4^th^, and 5^th^ instar caterpillars divided by the total number of milkweed examined at each site. After excluding sites where no larvae were detected (and hence there is no possibility for density-dependent effects), Lindsey et al. [15] calculated means and standard errors among three breeding regions and three time periods throughout the breeding season. Time periods during the breeding season were designated as early (before June 1^st^), middle (June 1^st^ to July 31^st^), and late (after July 31^st^) and the regions were designated as South (Texas, Georgia, North Carolina, Virginia and Tennessee), Midwest (Minnesota, Wisconsin, Michigan, Iowa, Indiana, Missouri, Ohio and Nebraska), and Northeast (Vermont, Maine, New York, New Jersey, Pennsylvania, District of Columbia and Ontario) following previously described patterns of population movement over successive generations throughout the breeding season [11]–[13], [28]. Analysis of variance of these data by Lindsey et al. [15] found a significant interaction of region and breeding phase on larval density (F_4,627_ = 2.82, P = 0.024) emphasising the complex spatio-temporal dynamics of larval density distribution in the monarch butterfly.

The densities presented by Lindsey et al. [15] could result in conservative estimates of the strength of density dependence because they ignored the potentially negative influence of extreme densities on larval survival. We therefore compiled published records of site-specific egg and larval field densities in the literature [11], [25], [26], and also applied our experimental results to these data, which allowed us to assess the relative importance of excluding extreme densities in the Lindsey et al. [15] dataset. As before, we excluded records with zero counts and assigned each record to a region and breeding phase following Lindsey et al. [15]. The data set only allowed a comparison to the early breeding phase in the southern region of Lindsey et al. [15] data. We calculated density as the number of large larvae (3^rd^, 4^th^ and 5^th^ instars), eggs, and the sum of all eggs and larvae (all instars) per milkweed plant and calculated the strength of density dependence for each.

### Modeling and Statistics

To test for a density-dependent reduction in egg laying and larval survival rates, we regressed the proportionate reductions in egg laying and survival on a logarithmic scale, known as killing values (k-value; [29], [30]), against log-transformed density. Larger k-values indicate an increasingly negative effect of density and a significant result (α = 0.05) indicates a density-dependent effect of intraspecific competition [30]. For egg laying rate, we calculated the k-value as the log of the mean per capita egg-laying rate at the lowest density (74 eggs per female) divided by the per capita number of eggs laid during the trial. Both the amount of cloud cover [24] and an individual’s age [20] are known to influence laying rates in monarchs. However, neither cloud cover (P = 0.68) nor a quadratic function of age (P = 0.09), influenced egg laying rate so we used a linear regression with density as the only explanatory variable. To test for a density-dependent effect of intraspecific competition on larval survival we calculated the k-value as the negative log of the number of butterflies that eclosed divided by the initial number of eggs [30] and regressed this against the starting egg density using a linear regression.

We used a general linear model of the percent remaining milkweed food resources at the end of the experiment against larval density to test if our results were driven by intraspecific competition for limited resources. To determine differences in growth rates, we used larval length and mass (both log-transformed) as response variables and included both density and age as explanatory variables to control for the strong effect of age on growth. We used age in days rather than in day-degrees [31] because we maintained the glasshouse under consistent temperature conditions throughout the experiment. We compared pupal length and mass, and, for adults that were not deformed after eclosion (e.g. from falling), forewing length and body mass. For the adult comparisons, we included sex in the model to control for known differences in size between males and females [15]. We measured development time in two ways: as the difference in the number of days from the egg being laid until either pupation or adult eclosion.

To test the effects of density on larval growth, we used linear mixed-effect models from the ‘lme4’ package [32] and for development time we used Cox proportional hazard models from the ‘coxme’ package [33] in program R [34]. Replicate was included as a random effect in all analyses (all P<0.0001). The statistical significance of growth on density was determined using likelihood ratio test that compared the change in residual deviance of a reduced model that excluded density to a chi-square distribution. We used density (log-transformed) in all comparisons with a continuous response variable.

We fit a general linear model to the experimental proportional survival of larvae to eclosion given initial egg density using a quasibinomial error structure. The y-intercept of this model was <1.0 (i.e. <100% survival) and was considered the density independent mortality rate that can arise in the absence of parasites [35] and predators [36] due to factors such as cardiac glycoside toxicity and amount of latex that can mire larvae [37].

To model the effect of variation in natural larval density on changes in monarch population size, we extracted the mean and standard error of larval density from Lindsey et al. [15] using the ‘digitize’ package in program R [38]. For each breeding phase and region, we randomly selected 100 density estimates from a normal distribution using the mean and 95% confidence interval of larval density and multiplied it by a randomly selected linear survival function using the slope and the 95% confidence interval of the parameters from our experiment. The strength of density dependence therefore included two sources of uncertainty, those of the mean density in wild populations and the linear survival function found in our experiment. To graph the possible proportional reduction of population size caused by density dependence over space and time we divided the predicted proportion of butterflies with and without density dependence and subtracted this value from one for each region and breeding phase.

Using the site-specific data we conducted two analyses. As above, we randomly selected 100 density estimates from a normal distribution using the mean and 95% confidence interval of density and multiplied it by a randomly selected slope and 95% confidence interval of the linear survival function to derive a mean and standard error estimate of the strength of density dependence. The other method applied a randomly chosen estimate of the linear function of the experiment to each site-specific density (n = 49) to calculate the strength of density dependence and conducted this procedure 100 times to generate a mean and standard error. We calculated density in three ways at each site as the number of large larvae, eggs, and the sum of eggs and all larvae per milkweed plant surveyed.

### Ethical Treatment of Animals

Butterflies for the stock population were captured under permit from the Ontario Ministry of Natural Resources (authorization number: 1057160). No animal care approval from the University of Guelph was required for this research.

## Results

### Egg Laying and Larval Density Experiments

Mean per capita egg laying rates in each replicate varied from 21 to 116 eggs per female (mean = 58.0, SD = 29.9) but density did not influence the per capita egg laying rates of females (F_1,18_ = 2.78, P = 0.113, r^2^ = 0.09). At the end of the larval experiment 98% of the milkweed food resources remained at the lowest density and 0% remained at the highest density (GLM: β = −22.51, SE = 1.70, t = −13.27, P<0.0001). Larval growth rates appeared linear until day 11 when most individuals began to form pupae. After accounting for the effect of age on larval size (length: β = 0.24, SE = 0.002, t = 119.5; mass: β = 0.58, SE = 0.007, t = 86.6), there was no reduction in length (β = 0.005, SE = 0.031, t = 0.19; χ2 = 0.002, df = 1, P = 0.97) or mass (β = −0.023, SE = 0.084, t = −0.27; χ2 = 0.04, df = 1, P = 0.84) of larvae with increasing density.

**Figure 1:**
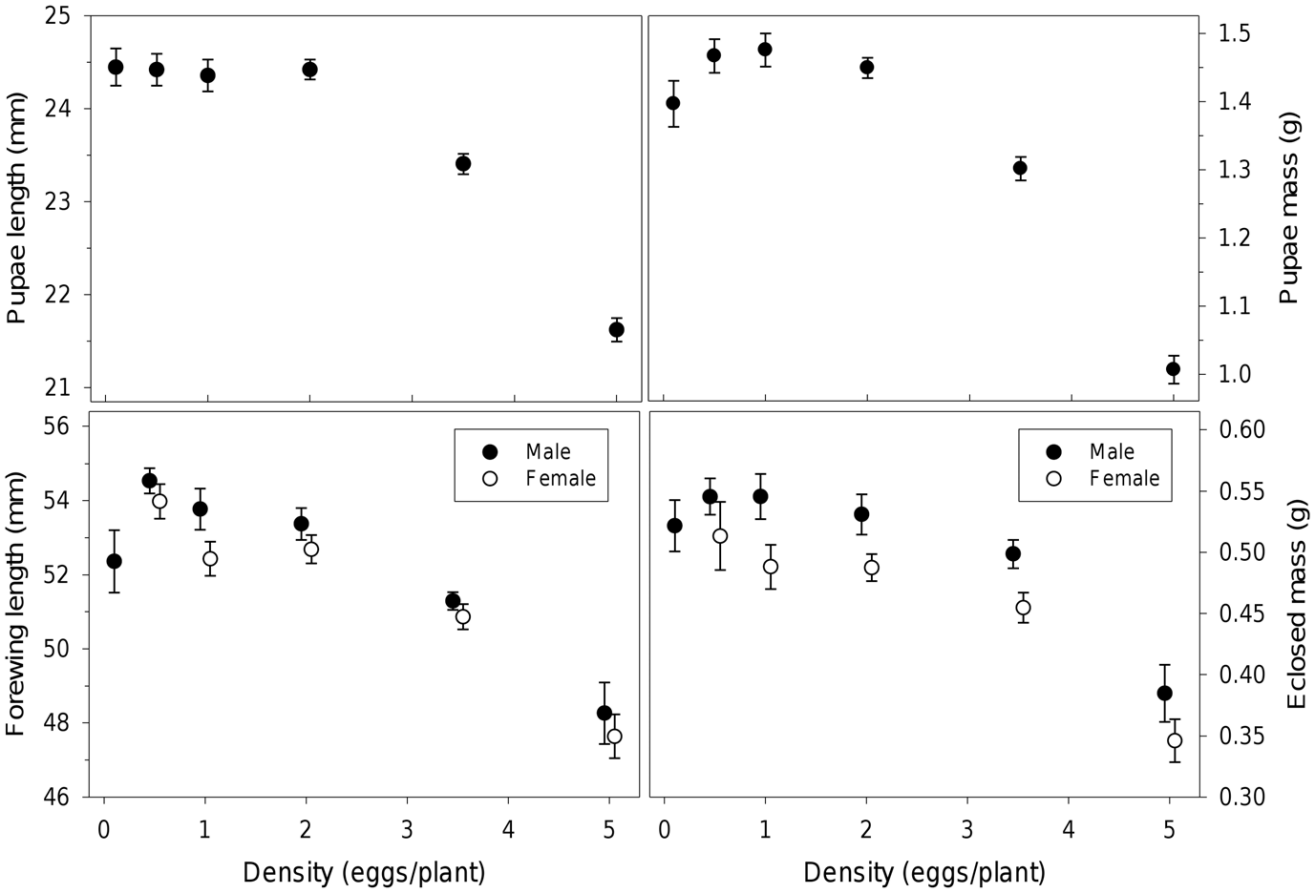
Effect of density on monarch butterfly length and mass. The mean (± SE) length (left) and mass (right) of pupae (top) and adult (bottom) monarch butterflies that were raised at different densities. Adult length is the length of the forewing. Both males (filled) and females (unfilled) are included in the plots of adults but sex only has a significant effect on eclosed mass (see text). No adult females were weighed or measured at the lowest density.

Although density had no influence on development time from egg to pupation (Z = −0.3, P = 0.76), pupae were both shorter (β = −0.62, SE = 0.168, t = −3.72; χ2 = 11.9, df = 1, P = 0.0005) and lighter (β = −0.088, SE = 0.0273, t = −3.23; χ2 = 9.32, df = 1, P = 0.002) at higher densities (Fig. 1). There was a negative relationship between development time from egg to eclosion and density: development was shorter at high density (Z = 2.09, P = 0.036) and females developed faster than males (Z = −2.32, P = 0.02). Eclosed adults had shorter wing lengths (β = −1.96, SE = 0.433, t = −4.53; χ2 = 16.2, df = 1, P<0.0001) at higher densities but there was no difference between males and females (β = 0.39, SE = 0.28, t = 1.36). Adults weighed less with increasing density (β = −0.049, SE = 0.014, t = −3.61; χ2 = 11.4, df = 1, P = 0.0007) and males weighed more than females (β = 0.03, SE = 0.01, t = 3.05; Fig. 1).

**Figure 2:**
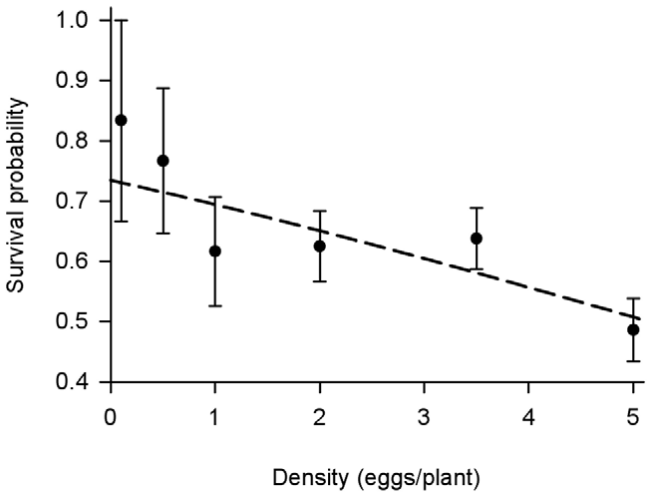
Density-dependent survival in monarch butterflies. The density-dependent effect of intraspecific competition presented as the mean (± SE) survival probability of monarch butterflies from egg to eclosion as a function on egg density per plant. There were six replicates per density treatment. The line represents the logit-link transformed survival function from a general linear model of survival using a quasibinomial error structure. The equation of the line is: 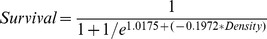
_._

The killing-value of density-dependent intraspecific competition increased at higher densities (F_1,33_ = 12.13, P = 0.001, r^2^ = 0.25) and competition was weakly contest-like (β = 0.15; [30]). When larval survival was regressed on density on an arithmetic scale using a binomial model there was a significant negative influence of density on the survival of eggs to adult butterflies (GLM: β = −0.008, SE = 0.0028, t = −2.80, P = 0.008). The estimate of density-independent survival rate from the model (i.e. the intercept) was 73.4%, with a 50.8% survival rate at the maximum density (Fig. 2).

### Applying Experimental Results to Natural Densities

**Figure 3:**
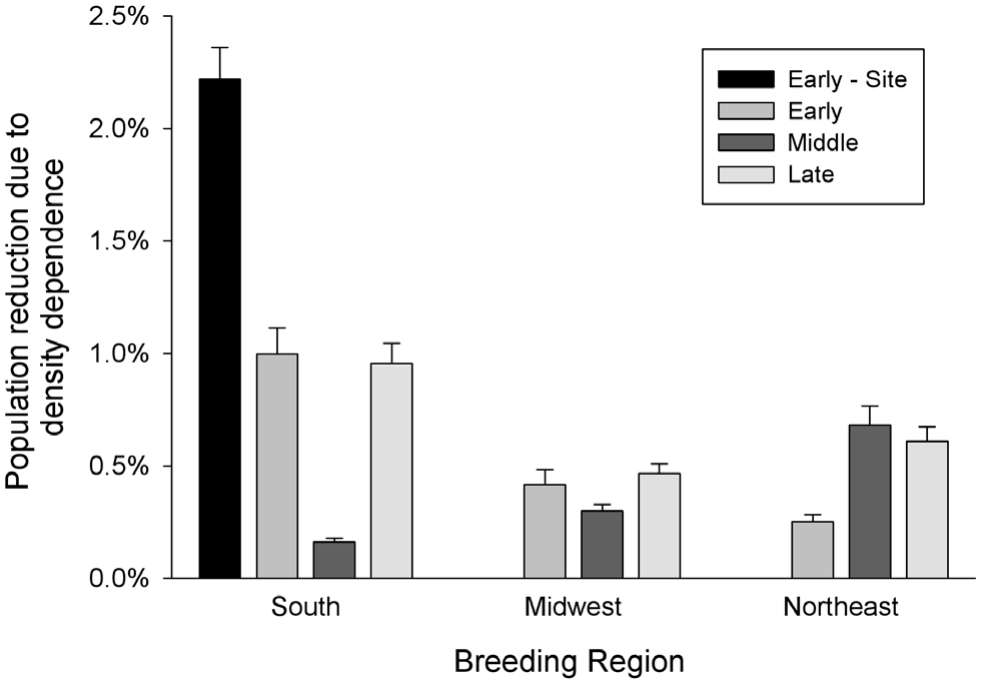
Predicted density-dependent population reduction of monarch butterflies during the breeding season in eastern North America. The estimated mean (± SE) percent population reduction of monarch butterflies throughout the breeding season across eastern North America caused by density-dependent intraspecific larval competition. The percent reduction is the quotient of the proportion of larvae predicted to eclose as adult butterflies with and without the effects of density dependence. The estimates are a product of the larval density data from Lindsey et al. [15] and the linear survival function from the density dependence experiment (Figure 2) and incorporates both the error in estimating mean density and the error associated with the slope parameter (see text). Estimates of the strength of density dependence are conservative because they are based on estimates of mean larval density for each region and breeding phase (see text) compared to the left-most bar (Early - Site) which uses data on larval density at each site to estimate the strength of density dependence (see Table 1).

Using the mean larval densities of monarch butterflies from eastern North America presented by Lindsey et al. [15], we applied the results of our density-dependent survival function to estimate the strength of density dependence across space and time. The strength of density-dependent intraspecific larval competition varied across location and phase of breeding (Fig. 3). Intraspecific competition in the South was highest in the early and late breeding phases and low in the middle phase. In the Northeast, competition was low in the early breeding phase and moderate in the middle and late phases. The Midwest had similar levels of competition during all breeding phases (Fig. 3). The predicted population reduction during the early breeding phase was four times higher in the South (1.0%) compared to the Northeast (0.25%), the opposite pattern occurred in the middle portion of the breeding season where the Northeast (0.68%) had the highest expected population reduction compared to the South (0.16%). Late in the breeding season, the three regions had similar estimated population reductions (Fig. 3).

The mean larval density in the early phase of the breeding season in the South was remarkably similar between the data sets: the mean density estimates of Lindsey et al. [15] was 0.231 larvae/plant (SE = 0.069) and the mean density of the site-specific data set [11], [25], [26] was 0.202 larvae/plant (SE = 0.041). Thus, the strength of density dependence using mean larval densities for the site-specific data (1.13%, SE = 0.11; Table 1) was directly comparable to Lindsey et al. ([15]; 1.00%, SE = 0.12; Fig. 3). When larval density was considered at each site individually, the population-level density-dependent mortality was 1.09% between estimates of percent reduction (Table 1). Therefore density dependence was about twice as strong when incorporating data with site-specific data compared to only using estimates of mean density (Fig. 3).

**Figure 4:**
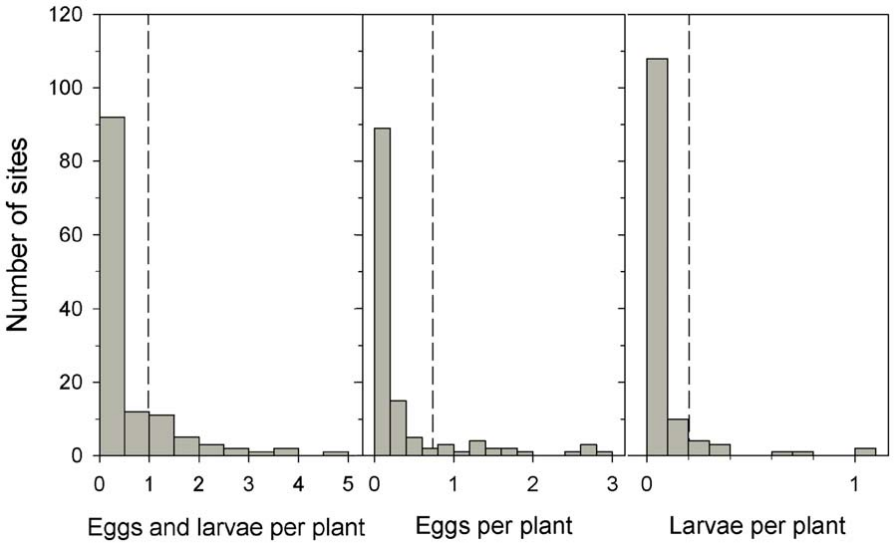
Distribution of three measures of monarch butterfly larval density. Histograms of the sum of egg and larval density (all instars), egg density, and large larvae (3^rd^, 4^th^, and 5^th^ instars) density from field surveys data compiled from the literature [11], [25], [26]. The predicted strength of density dependence is influenced by the different life-stages considered when estimating larval density and whether the density-dependent survival function is applied to the larval density at each site or to the mean population density (vertical dashed line). Using the mean population density to calculate the strength of density dependence excludes the extreme density values that occur regularly in the data set and results in conservative estimates of the strength of density dependence.

Using data from the early phase of the breeding season in the South [11], [25], [26], the strength of density dependence increased when we considered different life-stages to estimate monarch larval density as large larvae (mean = 0.202, SE = 0.041), eggs (mean = 0.736, SE = 0.120), or the sum of eggs and larvae (mean = 0.982, SE = 0.138; Fig. 4). Compared to large larvae density estimates, the strength of density dependence was about three times higher in eggs and four times higher in eggs and larvae (Table 1). For all three measures of density, the estimated strength of density dependence was twice as strong when considering density at each site data rather than using an estimated mean density value (Table 1).

**Table 1:** Predicted proportion reduction in population size in monarch butterflies caused by intraspecific density-dependent larval competition.

Population size reduction	Eggs andlarvae	Eggs	Largelarvae
Using mean density	5.31% (0.433)	3.96% (0.328)	1.13% (0.112)
Using site-specificdensity	10.87% (0.801)	8.69% (0.674)	2.22% (0.139)

Mean (standard error) percent population reduction caused by intraspecific density-dependent larval competition in monarch butterflies using two different methods of calculating the predicted proportion reduction population size. The three measures of density incorporate different life-stages. Densities of eggs and larvae include all larval instars whereas large larvae include only 3^rd^, 4^th^ and 5^th^ instars. Data come from published field surveys of milkweed and monarch larvae [11], [25], [26] and are for the early phase of the breeding season in the South (see text).

## Discussion

Our results provide evidence that larval mortality in monarch butterflies increases with larval density due to intraspecific competition for resources. Although our estimate of the strength of the density-dependent relationship was conservative, we also found that density-dependent competition has the potential to reduce the expected number of eclosing adult butterflies in the wild and, thus, potentially influence population growth rates in certain regions during specific portions of the breeding season. For the eastern North American population, southern areas during the early portion of the breeding season appear to have the highest levels of density-dependent mortality. The implication is that large numbers of overwintered butterflies from Mexico that return to lay eggs for the first generation [11] may experience higher levels of larval mortality and contribute significantly fewer offspring on a per capita basis than would smaller populations.

Based on our results from the egg laying experiment, we would not expect a reduction in the per capita number of eggs laid at high levels of competition among adult females. Instead, we found pupae and adults from the higher density treatments were smaller and lighter which implies that while larvae may develop normally, they are unable to carry-over the necessary resources to produce a normal-sized adult. In insects, smaller females have lower fecundity [4], [39] and laboratory experiments have found that smaller monarch butterflies had smaller eggs and a shorter lifespan that reduced lifetime fecundity compared to large butterflies [19]. Therefore, an indirect effect of density-dependent larval competition may be that larvae that experience high levels of competition also lay fewer eggs during their lifetime [4]. Ultimately, lower lifetime reproductive output mediated by resources from the larval stage could be one mechanism that leads to a density-dependent relationship with fecundity rather than adult egg laying rate via adult competition that we tested in this experiment.

Larvae in high density treatments did not have different growth rates suggesting that temperature-dependent development schedules are not likely overridden by the effects of density [31]. Although there was no relationship between density and development time from egg to pupation, development time from egg to adult was shortest for high density treatments. Our results contrast those of Lindsey et al. [15] and Atterholt and Solensky [40], who found that larval density did not influence development time from egg to eclosion. In our study, larvae were in direct competition for a finite amount of resources whereas subjects in Lindsey et al. [15] were provided enough food per individual to avoid density-related competition. Monarchs in Atterholt and Solensky [40] either experienced high density with no food competition or short-term food restriction but no competition for resources. Overall, we found that while larvae will maximize their feeding rates independent of density, adults that are smaller and in poorer body condition at eclosion arise from reduced development time during the pupal stage at higher density.

### The Strength of Density Dependence

When applied to field estimates, our results suggest that the strength of density dependence varies across location and time during the breeding period. This implies that, despite there being fewer expected generations at northern latitudes, females that migrate north to lay eggs may benefit by releasing their offspring from higher density-dependent competition in the early portion of the breeding season [11]. One hypothesis is therefore that the evolution of adult monarch butterfly migratory movements during the breeding season could be partially driven by density-dependent dispersal if adults can assess relative densities of other adult females or of immature densities. If so, then females should preferentially oviposit on plants without eggs, although this was not supported by one study [23]. Instead, the timing of migration is usually considered a response to milkweed emergence phenology [8] and the constraints of weather on movement patterns [11]. If so, then movement is independent of density and higher levels of larval competition result as a by-product of migratory behaviour when large numbers of adults move through geographic bottlenecks such as northern movement through Texas in the spring. We would expect a similar situation elsewhere if higher numbers of adults move through areas experiencing habitat loss. Given that reduction in milkweed is a major conservation concern [17], [41], this implies that conservation efforts integrated across the annual cycle could slightly increase recruitment simply by specifying where initial habitat restoration efforts should occur.

The strength of density dependence competition was influenced by which life-stages were considered when calculating intraspecific density and whether density was considered at a regional or local scale. Including eggs and all stages of larvae to calculate density resulted in population reductions that were about 5 times higher than those that considered only large larvae. In addition, larger larvae are likely to out-compete smaller larvae and larvae are known to cannibalize eggs [42], which imply size-dependent differences in competitive ability could further increase the strength of density dependence.

Our analysis of the strength of density dependence competition across space and time relied on published estimates of regional mean larval densities but, ideally, the analysis would have been done at a finer scale because larvae compete with conspecifics locally. The effect of density dependence at the site-level using a dataset of published surveys indicated the strength of density dependence was twice as strong as at the regional-level. However, larvae can only interact with conspecifics on a given plant and density estimates based on counts at the site-level usually include multiple plants that do not contain eggs or larvae [15]. The implication is that the small effects of density dependent competition seen throughout the breeding season at a geographic scale are predicted to influence local productivity at sites where larval density is high and larval dispersal is low. For example, milkweeds in agricultural landscapes occur at low density and are widely distributed [43] but these areas contain higher egg densities and thus contribute disproportionately more to monarch population growth compared to non-agricultural areas [14], [41]. Given the continuing reduction of milkweed resources in these productive habitats [43], [44], analyses of density-dependent mortality from competition which incorporate larval density and the spatial arrangement of milkweeds at the site level are likely to modify how these habitats are perceived to contribute to local monarch population size.

### The Mechanism by which Density Dependence May Operate

Invertebrate populations are generally considered to be limited by environmental stochasticity [45] with weather predicted to be the driving factor influencing vital rates [46], [47]. Monarch butterflies are subject to large-scale climate processes that influence vital rates such as the number of generations produced during the breeding season [11] and local weather conditions that influence mass-mortality events in Mexico [17]. These abiotic factors influence population growth stochastically. On the other hand, a variety of biotic factors are known to influence larval survival [14], [18], [35]–[37], [42], [48]. Predators [18], [36] and parasites [35], [48], in particular, are thought to strongly limit population growth rates. While most of these previous studies have measured vital rates independent of conspecific density, factors such as predation and parasitism could regulate monarch population growth if they operate in a density-dependent manner.

Assessing multiple density-dependent factors is difficult because for each mechanism it requires quantifying the strength of the effect and how it is predicted to influence population growth. For monarch butterflies, there are few experiments using variable yet realistic conspecific density treatments that measure the strength of these effects on changes of per capita vital rates (k-value [30]). Since density dependence can operate simultaneously through multiple mechanisms [49], [50], having multiple models to describe density-dependent effects would allow assessment of which factors, by comparing k-values [29], are then the most likely to regulate population size. Once candidate factors have been identified, a further problem then exists that there are likely to be few existing data to characterize the mechanisms to estimate how these important biotic processes influence monarch populations across space and time to understand year-round population dynamics.

Previous work on butterflies has found density-dependent effects during different stages of the life cycle could have a large influence on regulating population growth [4]–[6]. Other studies have measured and modeled density-dependent relationships of egg laying and predation rates of monarch butterflies on both the breeding [23] and wintering grounds [51]. Drury and Dwyer [23] found equivocal results of negative density dependence on laying rates and predation rates but were unable to explain natural variation in egg densities found in the wild. Calvert et al. [51] found a positive relationship between overwintering colony size and survival suggesting inverse density dependence. The results from these studies therefore do not provide a clear understanding of the strength or possible mechanism by which density dependence may operate in monarch butterflies.

Our study is the first to link experimental results of a density-dependent reduction in vital rates to natural observed monarch densities during the breeding season across North America. Density-dependent intraspecific larval competition is not thought to influence monarch population dynamics because larvae occur at relatively low density but theoretical arguments suggest that density dependence in insects is strongest at densities far below carrying capacity where selection promotes individuals that minimize density [2]. Hence, adults distributing eggs that result in low densities could therefore be an evolved response to density-dependent effects rather than an explanation for the relationship not occurring. Our application of experimental results to natural densities observed in the wild is predicated on a similar response between larvae in enclosures and larvae in the wild, particularly with respect to larval dispersal under increasing density. However, our intent was not to assert that intraspecific competition is the only mechanism by which density dependence can operate, rather that density dependence can spatially and temporally vary in migratory species, is dependent on life history, and is likely to influence conservation decisions because it links population-level responses to geographic landscapes.
